# Involvement of palms and soles in patients with autoimmune bullous diseases: a comparative analysis of a diagnostically relevant localization

**DOI:** 10.3389/fimmu.2023.1227855

**Published:** 2023-10-10

**Authors:** Magdalena Jałowska, Maciej Spałek, Monika Bowszyc-Dmochowska, Justyna Gornowicz- Porowska, Marian Dmochowski

**Affiliations:** ^1^ Autoimmune Blistering Dermatoses Section, Department of Dermatology, Poznan University of Medical Sciences, Poznań, Poland; ^2^ Cutaneous Histopathology and Immunopathology Section, Poznan University of Medical Sciences, Poznań, Poland; ^3^ Department and Division of Practical Cosmetology and Skin Diseases Prophylaxis, Poznan University of Medical Sciences, Poznań, Poland

**Keywords:** palms, soles, autoimmune blistering dermatoses, pemphigus, pemphigoid

## Abstract

**Introduction:**

The involvement of palms and soles is variable among disease entities belonging to autoimmune bullous diseases (AIBD). We present our own clinical-laboratory experience concerning presentations of skin lesions on palms and soles in the pemphigus diseases group, pemphigoid diseases group, epidermolysis bullosa acquisita (EBA), and lichen planus pemphigoides (LPP) and discuss the pertinent literature.

**Methods:**

Lesions on palms and soles were assessed retrospectively on the basis of just photographic archives from the beginning of 2014 to March 2023. We comparatively evaluated 462 Slavic patients with AIBD.

**Results:**

Palmoplantar involvement was observed in only 21 patients with AIBD (12 females and 9 males). There was no statistically significant difference between palmoplantar involvement in the pemphigus diseases group compared to the pemphigoid diseases group and no statistically significant difference between the pemphigus diseases group compared to the subepithelial AIBD.

**Discussion:**

Nevertheless, particularly in LPP and EBA, and occasionally in pemphigus diseases and pemphigoid diseases groups of AIBD, localization on palms and soles may be diagnostically important at the clinical level.

## Introduction

Autoimmune bullous diseases (AIBD) are a heterogeneous group of dermatoses associated with autoimmunity directed against desmosomal structural proteins (pemphigus diseases group) or against dermal–epidermal junction structural proteins, namely, pemphigoid diseases group and epidermolysis bullosa acquisita (EBA), or with autoimmunity to enzymes-epidermal/tissue transglutaminases (dermatitis herpetiformis), clinically characterized by blisters and erosions on the skin and/or mucous membranes (1).

Pemphigus is a group of chronic, rare, potentially life-threatening AIBD of the skin and/or mucous membranes characterized most commonly by autoimmunity against desmoglein (DSG) proteins, namely, DSG3 alone, DSG1 alone, or both DSG3/DSG1, but other antibodies may be involved in immunization (2). Pemphigus vulgaris (PV), pemphigus foliaceus (PF), and paraneoplastic pemphigus (PNP) are the main subgroups of pemphigus (2). The skin lesions in PV typically can be found on the scalp, face, trunk, axilla, groin, and also pressure points (2). Oral and other mucosal lesions are seen in 80 to 90% of cases and may represent initial presentations of PV (2). PV “likes” areas adjacent to natural body orifices (1, 2).

The pemphigoid diseases group is the most common group of AIBD associated with tissue-bound and circulating autoantibodies most often directed against hemidesmosmal transmembrane protein (BP180, BPAG2, or type XVII collagen) and/or hemidesmosomal intracytoplasmic protein (BP230, BPAG1e—epithelial isoform); also, other antibodies may be involved in immunization (3). Pruritic tense subepidermal blisters usually develop in flexural areas that are proximally located, such as the axilla and the groin, inner thighs, and abdomen. The blisters can be localized or widespread (3).

Lichen planus pemphigoides (LPP) combines the features of lichen planus and bullous pemphigoid (BP). The hallmarks of this disease are lichen planus lesions with numerous bullae which develop in the context of autoantibodies targeting BP180 and/or BP230. LPP lesions are predominantly found on the extremities (4).

Epidermolysis bullosa acquisita (EBA) is a rare, clinically heterogenous, subepithelial AIBD which presents vesicle and bullae formation on the skin, and erosions on the mucous membranes associated autoantibodies to the VII collagen (coll7) (5). The disease has two main clinical forms: inflammatory and mechanobullous (classical or non-inflammatory). Patients with mechanobullous EBA develop non-inflammatory bullae and erosions at sites of trauma, while patients with the non-mechanobullous type develop inflammatory lesions which often mimic other blistering conditions including BP, linear IgA bullous disease, and mucous membrane pemphigoid (5). In the mechanobullous disease variant, skin fragility and blister formation occur with residual scarring and milia production on extensor surfaces of the skin and sites prone to trauma (6).

The acrally located blisters can present in one of two clinical scenarios. The first scenario occurs when the blisters can be localized only to the palms and soles with no additional bullous lesions. The second scenario involves the acral blisters preceding the development of blisters at other locations (7). Palmoplantar involvement is relatively common in patients with EBA and LPP, whereas palmoplantar involvement in the pemphigoid diseases group (excluding LPP) and pemphigus diseases group was very rarely described, and we do not know the exact frequency of occupation of these areas.

## Aims

The aim of this study was to comparatively analyze the involvement of the palms and soles in AIBD mediated by autoimmunity to predominantly structural proteins in ethnic Poles in a setting of a single university referral center.

## Materials and methods

This study was conducted in a setting of a Central European university dermatology department on 462 Slavic patients (males and females) with AIBD: pemphigoid diseases group (autoimmunity to BP180 BP230 or both), pemphigus diseases group (autoimmunity to DSG1, DSG3, or both), EBA (autoimmunity to coll7), and LPP (autoimmunity to BP180). We separated the group with LPP from the pemphigoid diseases group because, in this entity, involvement of the extremities is relatively very common. The group of patients studied was retrospectively analyzed for the presence of skin lesions on palms and soles. The lesions on palms and soles were assessed on the basis of just photographic archives in the period from the beginning of 2014 to March 2023. In this study, the involvement of palms and soles was analyzed jointly, as their microscopic features are similar. The AIBD appropriate treatment-naïve patients evaluated were as follows: 347 patients of the pemphigoid diseases group, 104 of the pemphigus diseases group, 8 with EBA, and 3 with LPP. The diagnoses of AIBD were based on the combination of clinical data, and DIF of perilesional tissue for evaluation of IgG, IgG1, IgG4,IgA, IgM, and C3 deposits, as well as monoanalyte ELISAs with DSG1, DSG3, BP180, and BP230 (initially MBL, Japan later Euroimmun, Germany) or multiplex ELISA with six different antigens (BP180, BP230, DSG1, DSG3, envoplakin, coll7) (Euroimmun, Germany) corroborated in some cases by the histopathological H+E examination, 6-substrate mosaic indirect immunofluorescence (IIF) (Euroimmun, Germany) or mosaic IIF with laminin 332 (Euroimmun Germany).

The monoanalyte ELISA kits manufactured by Euroimmun Germany utilized DSG1 and DSG3 recombinant proteins consisting of five subdomains of the extracellular domain of those molecules, as well as BP180 recombinant protein consisting of four copies of its most immunogenic domain called NC16A and BP230 recombinant protein consisting of a duplicated fragment of its C-terminal globular domain (manufacturer’s cutoff values of 20 RU/ml). The recombinant DSG1, DSG3, BP180, and BP230 used in the multiplex ELISA system were the same as those in the monoanalyte ELISA kits. In multiplex ELISA, each antigen was coated in a separate well and a semiquantitative evaluation was carried out with the manufacturer’s cutoff ratio of 1. All measurements were made in the ELISA plate readers (Asys Expert 96 or Ledetect 96) equipped with MikroWin 2000 software by a single operator following the manufacturer’s instructions.

### Statistical analysis

The Z test for two independent proportions was used for our calculations. Statistical analysis was performed using the PQStat 1.8.2 program (PQStat Software, Plewiska, Poland). Statistical significance was α = 0.05. A result was deemed statistically significant if p < α.

## Results

### Diagnoses and statistics

**Figure 1:**
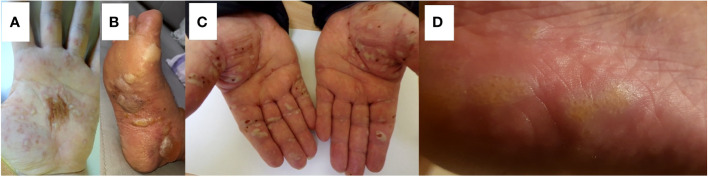
Representative AIBD patients showing palmar or plantar involvement. **(A)** A 29-year-old male with mucocutaneous pemphigus vulgaris apparently induced by escitalopram treatment for depression having palmar lesions. Direct immunofluorescence examination—IgG4 (++) pemphigus epidermal deposits; multiplex ELISA—increased levels of IgG antibodies against DSG1 (1.8) and against DSG3 (4.4) (**Table 1**, #2). **(B)** A 71-year-old female with bullous pemphigoid showing plantar lesions. Direct immunofluorescence examination—deposits of IgG4 (+), C3 (++) with a pattern along the dermal–epidermal junction; multiplex ELISA—increased levels of IgG antibodies against BP180 (8.50) and against BP230 (4.98) (**Table 1**, #4). **(C)** A 73-year-old male with epidermolysis bullosa acquisita which developed following the initial diagnosis of Brunsting-Perry pemphigoid showing palmar lesions. Direct immunofluorescence examination—deposits of IgM (+), IgG (+/−), IgG1 (+), IgG4 (+++), and C3 (++) with a pattern along the dermal–epidermal junction; multiplex ELISA—increased level (4.54) of anti-coll7 IgG antibodies (**Table 1**, #9). **(D)** An 80-year-old female with lichen planus pemphigoides following nivolumab treatment for melanoma showing plantar lesions. Direct immunofluorescence examination—deposits of IgA, IgM, IgG, IgG1, IgG4, and C3 not found; multiplex ELISA—increased level (1.73) of anti-BP180 IgG antibodies (**Table 1**, #21).

The mean age of the patients with AIBD and palmoplantar involvement was 63 years. Palmoplantar involvement was observed in 21 of 462 patients with AIBD (11 females and 9 males). None of our patients had isolated skin lesions on the palms and soles. All of our patients had generalized blistering lesions with involvement of the palms and soles. Skin lesions on the palm and sole area were present in two patients in the pemphigus diseases group (1.92%), 10 in the pemphigoid diseases group (2.88%), 6 EBA patients (75%), and all patients with LPP (100%). The proportion of lesions on the palms and soles among the pemphigus diseases group (2 of 104, 1.92%) did not differ statistically significantly from the proportion in the subepithelial AIBD (pemphigoid diseases group, EBA and LPP patients) (19 of 357, 5.32%) (p = 0.269). The proportion of lesions on the palms and soles in patients from the pemphigus diseases group (1.92%) was not statistically significantly different from the proportion in patients from the pemphigoid diseases group (10 of 347, 2.88%) (p = 0.853). The LPP and EBA groups were too small to be separately reliably statistically evaluated. Clinical characteristics of AIBD patients with palm and sole involvement are shown in **Table 1** and **Figure 1**.

**Table 1:** Clinical characteristics of AIBD patients with palm and sole involvement.

#	Age/ gender	Diagnosis		Treatment	Response to treatment		
1	a/M	Pemphigoid diseases group/BP		GCS (M) tGCS	CR		
2	y/M	Pemphigus diseases group/PV		GCS (M) tGCS IVIG	CR		
3	a/F	EBA		GCS (M) tGCS DDS	CR		
4	o/F	Pemphigoid diseases group/BP		GCS (M) tGCS	CR		
5	a/F	Pemphigus diseases group/PF		iGCS (psychotic symptoms) GCS (M) tGCS CTX (toxic liver damage) DDS RTX is scheduled	PR		
6	o/M	Pemphigoid diseases group/BP		DXC GCS (M) DDS CTX MTX	CR		
7	o/F	EBA		GCS (M) tGCS DDS AZA MTX CTX RTX IVIG TP	PR, then lost to follow-up		
8	i/M	Pemphigoid diseases group/BP		iGCS IVIG GCS (P)	CR		
9	o/M	EBA		iGCS	CR		
10	y/F	Pemphigoid diseases group/pemphigoid gestationis		GCS (P)	CR		
11	o/F	EBA		GCS (M) tGCS	Initially PR, then lost to follow-up		
12	o/F	EBA		GCS (M) tGCS DXC	CR		
13	a/M	LPP		GCS (M) tGCS	CR		
14	o/M	Pemphigoid diseases group/BP		DXC DDS GCS (M) IVIG	CR		
15	o/F	Pemphigoid diseases group/BP		GCS (M) tGCS CTX DXC	CR	
16	o/F	Pemphigoid diseases group/BP		GCS (M) tGCS	CR	
17	a/F	EBA		GCS (M) tGCS	CR	
18	o/M	Pemphigoid diseases group/BP		DXC GCS (M) tGCS	CR	
19	o/M	LPP		GCS (M) tGCS	CR		
20	o/M	Pemphigoid diseases group/BP		GCS (M) tGCS	CR		
21	o/F	LPP		tGCS	CR

Age range: i—infancy under 1 yr.; ch—childhood, 1–17 yrs.; y—youth 18–35 yrs.; a—adulthood, 36–64 yrs.; o—old age above 65 yrs.

tGCS, topical glucocorticosteroids; GCS, oral glucocorticosteroids: GCS (M) methylprednisolone, GCS (P) prednisone; iGCS, intravenous glucocorticosteroids pulses-methylprednisolone; AZA, azathioprine; MTX, methotrexate; CTX, cyclophosphamide; CSA, cyclosporine; DDS, dapsone; IVIG, intravenous immunoglobulin; DXC, doxycycline; RTX, rituximab; TP, therapeutic plasmapheresis.

A complete remission (CR) (8).

A partial remission (PR) (8).

### Response to therapy

All but three patients with involvement of palms/soles exhibited favorable responses to the first-line therapeutic interventions involving systemic glucocorticosteroids (GCS) or potent topical GCS (**Table 1**, #21; this patient with LPP declined the systemic GCS treatment). One patient with mucocutaneous PV necessitated sequential therapy, which included oral GCS and four intravenous immunoglobulin infusions to achieve remission (**Table 1**, #2). Similarly, for another patient with the classic/mechanobullous form of EBA, a complex therapeutic approach was employed. This encompassed sequential administration of oral GCS, dapsone, azathioprine, methotrexate, cyclophosphamide, cyclosporine, five sessions of plasmapheresis, and 12 intravenous immunoglobulin infusions. Eventually, two infusions of rituximab (each 1 g) and intravenous GCS were used to bring the disease under partial control (**Table 1**, #7). In one patient with PF, a favorable response to therapy was achieved by adding orally administered cyclophosphamide to the initial intravenous and then oral GCS treatment (**Table 1**, #5). However, this intervention led to transient toxic liver damage, prompting the discontinuation of cyclophosphamide. Following the normalization of liver function parameters, dapsone at a daily dose of 50 mg was introduced. Despite these efforts, the cutaneous lesions continued to relapse, necessitating further treatment with intravenous GCS, which resulted in a partial reduction of skin lesions. Unfortunately, psychotic symptoms occurred. The administration of rituximab is scheduled.

## Discussion

The single-center nature of this study, retrospective evaluation of just photographic archives available, and a small number of EBA and LPP cases are limitations of our research design. Importantly, diagnosing patients was done with biochemical–molecular techniques, not merely imaging ones.

The involvement of palms and soles is variable among disease entities belonging to AIBD. It was far more common in our EBA and LPP patients compared to the pemphigus diseases and pemphigoid diseases groups. Nevertheless, the proportions of patients with the involvement of palms and soles in the pemphigus diseases and pemphigoid diseases groups were small and did not differ statistically significantly.

Mechanical stress pressure could have triggered blisters in patients at clinically non-typical body sites (2). Another reason for the appearance of skin lesions on palms and soles could be localized trauma-induced koebnerization (9). The Koebner phenomenon (KP) may be classified into several different groups including true koebnerization (e.g., vitiligo, lichen planus, psoriasis), pseudo-koebnerization (occurs by seeding of infectious agents into traumatized skin), and localized trauma-induced koebnerization (10). Trauma-induced pemphigus has been reported after surgery, radiation, excessive sun exposure, and burns. In these cases, blister and bullae formation at the sites of injury were caused by localized exposure to self-antigens (10, 11). Rashid et al. described a patient with blisters which initially appeared around a surgical incision, 2 weeks following an appendectomy (12). Duick et al. presented a case of pemphigus that started in a Mohs surgical wound after excision of a squamous cell carcinoma (SCC) from a 49-year-old woman (13). Jetter et al. described pemphigus vegetans after cryosurgery for actinic keratosis (AKs) at the temple and forehead (14). In addition, minor procedures such as periodontal procedures, electrosurgery, Mantoux test, or laser surgery could also trigger pemphigus (10, 15). The explanation might be that epidermal injury may expose DSG 1 and 3 and lead to new autoantibody formation in genetically susceptible patients or to activation of preexisting antibodies already present in low (subclinical) titers (14, 16). Mechanical trauma and radiation are known also to trigger localized BP (17). Burns, phototherapy, X-ray irradiation, surgical incision sites, lymphedema, fistulas, and ostomies were described as an inducer of BP (10).

In the literature, we found a description of unilateral, localized BP in a patient with chronic venous stasis (18). The physical and immunologic changes associated with chronic venous stasis may have predisposed the patient to autoantigen presentation, generation of autoreactive T cells, and subsequent autoantibody-producing B cells (18). The anti-BP230^+^ and anti-BP180^−^ immunologic profile observed in this patient has also been described in the case of a patient who had localized BP in a bandlike pattern on the bilateral lower extremities (18). Furthermore, six in a series of eight patients with localized BP were found to be anti-BP230^+^ and anti-BP180^−^ in one study (19). Epidermal injury or inflammation is thought to uncover the antigen in predisposed individuals and results in new koebnerization lesions (20). Antibodies to the BP180 transmembrane protein are the primary mediators of BP. The intracellular location of BP230 in a hemidesmosomal plaque presumably makes it less prone to autoantibody attack. Autoantibodies directed against BP230 may result in a secondary phenomenon that is important in the pathogenesis of epitope spreading. However, in our patients with palmoplantar involvement in the pemphigoid diseases group, there was no patient with isolated anti-BP230 antibodies. In the pemphigoid diseases group, 50% of patients had anti-BP180 antibodies concomitant with antibodies against BP230, whereas in the remaining 50% of patients, only anti-BP180 antibodies were detected.

In the mechanobullous form of EBA, skin fragility and vesiculobullous lesions occur in areas that are more subject to pressure and trauma, especially the extensor surfaces of the acral regions (hands, feet, elbows, knees, and pretibial region) (5). We found numerous descriptions of the location of EBA on the palms and soles (21–23). They appear soon, or at most a few hours after trauma to the skin, which can be minimal (5). In the inflammatory form, lesions occur throughout the skin, not only in areas most often subject to trauma, and skin fragility is not so important. In LPP, lesions are predominantly found on the extremities (4, 24, 25), and involvement of palms and soles occurs more frequently in children (25). All our patients with LPP had palmoplantar involvement.

Another explanation for the location of skin lesions on the palms and soles may be the dyshidrotic form of AIBD. Dyshidrosiform BP, a less stereotypical form of BP, usually presents with pruritic blisters in elderly individuals; the hemorrhagic or purpuric lesions on the palms and soles can be the only manifestation of the disease (7). Dyshidrosiform pemphigus, however, is rarely seen (26). Palmoplantar involvement in PV is a poor prognostic indicator, and palm involvement was associated with aggressive behavior of the PV. The exact pathogenesis of dyshidrosiform pemphigus is unknown (26). Nevertheless, there are data showing that murine DSG1 is expressed in the eccrine glands; therefore, it may be an explanation for this clinical feature (27). A similar prognostic importance of palm involvement is also reported by Vaishnani et al. (28). Borradori et al. have reported PV presenting as pompholyx of the left foot (29). Bolling et al. (30) have reported a unique case of palmoplantar keratoderma and a pemphigus-like immunobullous disorder, with an antibody against desmocollin 3 (not against DSG 3). However, this case showed no classical dyshidrosiform involvement.

Long-lasting and recurring bullous skin lesions with palmoplantar involvement always require a detailed differential diagnosis. The morphology of palmar and plantar bullous lesions can mimic numerous conditions including dyshidrosiform dermatitis, allergic or irritant dermatitis, cutaneous T-cell lymphoma (vesicular palmoplantar variant), porphyria cutanea tarda, dystrophic EB, dermatophyte infection, erythema multiforme, impetigo bullous, lichen planus bullous, scabies bullous, bullous systemic lupus erythematosus, Sweet’s syndrome, herpes simplex (HSV) type 1 and 2, and infectious diseases with blisters (2). Indeed, the possibility of the AIBD should be initially considered also in those individuals in whom blisters occur solely on their palms and soles (2).

## Conclusion

Putting aside the frequency issues, the AIBD should be kept in mind in the differential diagnosis at the clinical level of bullous lesions on the palm and soles. The involvement of palms and soles is frequent in LPP and EBA, whereas it is occasional in pemphigus diseases and pemphigoid diseases groups. In patients, showing bullous palmoplantar lesions thorough immunopathological examinations as a part of differentiating laboratory workup may be required for the definitive AIBD diagnosis (31).

## Data availability statement

The original contributions presented in the study are included in the article/supplementary material. Further inquiries can be directed to the corresponding author.

## Ethics statement

This study protocol was reviewed and approved by the local Polish Ethical Committee of the Poznan University of Medical Sciences, approval number (560/15). The patients/participants provided their written informed consent to participate in this study. Written informed consent was obtained from the individuals and the minor’s next of kin for the publication of any potentially identifiable images or data included in this article.

## Author contributions

Conceptualization MD; methodology MJ; software MJ, MD, JG-P; validation JG-P, MB-D; MS formal analysis MJ, MB-D; investigation MD; resources MD, MJ, data curation MJ, JG-P; writing—original draft preparation MJ; writing—review and editing MJ, MD; visualization MD; supervision MD project administration MJ, MB-D, JG-P. All authors have read and agreed to the published version of the manuscript.
